# Effect of Counterion Valence on Conformational Behavior of Spherical Polyelectrolyte Brushes Confined between Two Parallel Walls

**DOI:** 10.3390/polym10040363

**Published:** 2018-03-24

**Authors:** Lujuan Li, Qianqian Cao, Chuncheng Zuo

**Affiliations:** 1 College of Mechanical and Electrical Engineering, Jiaxing University, Jiaxing 314001, China; zuocc@jlu.edu.cn; 2 China-Australia Institute for Advanced Materials and Manufacturing, Jiaxing University, Jiaxing 314001, China

**Keywords:** polyelectrolyte brushes, molecular dynamics simulations, confinement, conformational behavior

## Abstract

We study the conformational behavior of spherical polyelectrolyte brushes in the presence of monovalent and trivalent counterions in a confined environment. The confinement is exerted by two parallel walls on the brushes. The enhancement of the confinement induces the extension of grafted chains. For the monovalent case, the increase of the charge fraction leads to extended brush conformation for different slit width (distance between two walls) but collapsed brush in the presence of trivalent counterions is observed. The confinement does not affect electrostatic correlation between trivalent counterions and charged monomers. However, it was found that narrow slit width contributes to stronger electrostatic correlation for the monovalent case. This is because more monovalent counterions are inside the brush at strong confinement, but almost all trivalent counterions are trapped into the brush independently of the slit width. The diffusion of counterions under the confinement is related to the electrostatic correlation. Our simulations also reveal that the brush thickness depends on the slit width nonlinearly.

## 1. Introduction

When polyelectrolyte chains are grafted densely on a substrate surface, also known polyelectrolyte brushes, the brushes exhibit rich conformational behavior due to the long-range electrostatic interaction [1,2,3,4]. Their chemical tailoring and well responsive properties lead to a wide range of applications. To obtain an insight into the structures and dynamics of these systems, theoretical studies of polyelectrolyte brushes in solution based on self-consistent field theories [5,6,7] and scaling laws [8,9,10] were performed. Other methods, such as molecular theories [11,12,13], including more chain structure details, can provide more accurate results.

During the past decade, molecular simulations have been extensively used to investigate the conformational behavior of polyelectrolyte brushes [14,15,16,17,18,19,20,21,22]. Seidel et al. first employed coarse-grained computer simulations to study planar polyelectrolyte brushes [14]. Their simulations reveal that the thickness of strongly charged polyelectrolyte brushes is linearly proportional to the chain length and the grafting density, and the counterion distribution is strongly inhomogeneous due to strong correlation between polyelectrolyte chains and counterions. At weak electrostatic interactions, the chains become strongly stretched, and a weak dependence of the brush thickness on the grafting density is observed contrary to the well-known scaling law for the osmotic brush regime. The structures of spherical polyelectrolyte brushes (SPBs) in the presence of multivalent counterions [20], oppositely charged linear polyelectrolytes [21], and oppositely charged surfactants [22], have been studied through computer simulations. The brushes adopt various conformational behavior depending on different oppositely charged components and their concentrations. Except for equilibrium brush conformations, molecular simulation works on the polyelectrolyte brushes in external fields, such as electric field [23,24,25], have been performed. The electric field can influence the fluid transport in channels coated with polyelectrolyte brushes through changing the conformations of the brushes.

Studying dynamics of polymers in confined domains has great significance in application and scientific research [26,27,28,29,30]. For example, confinement of denatured proteins in the cage model can accelerate folding compared with folding under bulk condition. It is also an important subject to investigate confinement dynamics of genomic double-stranded (ds) DNA in viral capsids with a diameter smaller than or comparable to the dsDNA persistence length. The confinement lowers the conformational entropy of the chain because of less number of conformational states available to the chain. The chain is forced to form energetically unfavorable conformations and exerts a pressure onto the confinement boundary. The confinement problem of linear polymers has been considered extensively in previous works, but related problem on polymers with complex chemical architecture, such as branched polymers and polymer brushes, has received much less attention. Star polymers confined in a nanoslit [31] and near a planar surface [32,33] have been studied through MD simulations. In this work, we study a single SPB in the presence of monovalent and trivalent counterions confined between two planes using MD simulations. The effect of the counterion valence on the conformational behavior of the SPB in the confined environment has not been studied so far. The remainder of the paper is organized as follows. In the next section we describe the simulation methods and system. Following that, the results are presented and discussed. Finally, conclusions are given in Section 4.

## 2. Simulation Model and Method 

The SPB considered in this work consists of a spherical core uniformly grafted with $N_{g}=40$ fully flexible polyelectrolyte chains, each containing $N_{gm}=32$ monomers. The grafting density is represented as $\rho_{g}=N_{g}/4\pi R^{2}$, where R=3σ is the radius of the core. The core of the SPB is fixed at the center of the simulation box. Each chain is charged by a fraction f in a periodical manner: every 1/f monomer carries a negative charge. To satisfy electric neutrality, $N_{i}=fN_{g}N_{gm}/Z$ counterions are added to the system. No additional salt is included. All particles are enclosed in a simulation box with a box length $L_{x}=L_{y}=160\sigma$ and the periodic boundary condition is applied in the x and y directions. Our simulation box is large enough to avoid short-range interactions of the SPB with its periodic images. The z direction is non-periodic and the z-directional box length $L_{z}$ (the slit width, namely the distance between two parallel walls) is tunable to study the conformational behavior of the SPB for different confinement cases.

The potential energies and their parameters used in the paper are analogous to those in our previous works on polyelectrolyte systems [22,34]. The short-range interaction between any two particles is modeled by the truncated-shifted Lennard-Jones (LJ) potential.

(1) $$U_{LJ}(r)=\{\begin{array}{ll} 4\varepsilon_{LJ}\left[{\left(\sigma /r\right)}^{12}-{\left(\sigma /r\right)}^{6}+1/4\right], & r<r_{c}\\ 0, & r\geq r_{c} \end{array}$$

where σ and $\varepsilon_{LJ}$ are the Lennard-Jones parameters. For all particle pairs, the cutoff distance is taken to be $r_{c}=2^{1/6}\sigma$, corresponding to a purely repulsive interaction between the particles. σ, m and $\varepsilon_{LJ}$ (for the interaction between polyelectrolyte monomers) are taken as the length, mass, and energy units, respectively. All other units are derived from these basic units, such as time unit $\tau ={\left(m\sigma^{2}/\varepsilon_{LJ}\right)}^{1/2}$ and temperature unit $T^{*}=\varepsilon_{LJ}/k_{B}$. The beads are coupled by a finitely extendable nonlinear elastic (FENE) potential [35].

(2) $$U_{bond}(r)=-(kR_{0}^{2}/2)\ln(1-r^{2}/R_{0}^{2})$$

where the maximum bond length is $R_{0}=1.5\sigma$ and the spring constant is given by $k=30\varepsilon_{LJ}/\sigma^{2}$. This choice of parameters gives an average bond length a=0.98σ. The combination of LJ and FENE potentials ensures that the constituent chains cannot pass through one another. One end of each polyelectrolyte chain is anchored onto the grafting surface or the core surface. All particles except for the grafted monomers interact repulsively with the core through the LJ potential with a shifted distance R. The electrostatic interaction between any two charged particles with charge valences $Z_{i}$ and $Z_{j}$, and separated by a distance $r_{ij}$ is modeled by the Coulomb potential.

(3) $$U_{Coul}(r_{ij})=k_{B}TZ_{i}Z_{j}\frac{\lambda_{B}}{r_{ij}}$$

where the Bjerrum length $\lambda_{B}=e^{2}/\left(4\pi \varepsilon_{0}\varepsilon_{r}k_{B}T\right)$ is the distance at which the electrostatic energy between two elementary charges is comparable in magnitude to the thermal energy $k_{B}T$. $\varepsilon_{0}$ and $\varepsilon_{r}$ are the vacuum permittivity and the dielectric constant of the solvent, respectively. The long-ranged Coulomb interactions is calculated using the particle-particle/particle-mesh (PPPM) algorithm, which maps the charge to a 3D mesh and uses fast Fourier transforms (FFTs) to solve Poisson's equation on the mesh. To calculate the Coulomb interaction of systems with a slab geometry which are periodic in the x- and y-direction and have a finite length in the z-direction, an empty volume with a height of $nL_{z}$ is inserted along the z-direction. For all runs, n=3 is taken. A correction term is also included into the modified PPPM algorithm [36]. The interaction between the walls and particles is modeled using an integrated LJ potential

(4) $$U_{wall}\left(\Delta z\right)=\frac{2\pi \varepsilon_{w}}{3}\left[\frac{2}{15}{\left(\frac{\sigma }{\Delta z}\right)}^{9}-{\left(\frac{\sigma }{\Delta z}\right)}^{3}\right]$$

where Δz is the distance of the particles from the wall, and $\varepsilon_{w}=0.1\varepsilon_{LJ}$.

The system temperature is held at $T=1.2T^{*}$ using a Langevin thermostat. The positions and velocities of the particles are solved using the velocity-Verlet algorithm. All simulations are conducted with a time step Δt=0.008τ. We run an equilibrium simulation for $2.0\times 10^{6}$ time steps. After achieving an equilibrium state, a production run of $2.5\times 10^{6}$ time steps is performed to obtain equilibrium properties.

## 3. Results and Discussion

### 3.1. Brush Conformation

**Figure 1:**
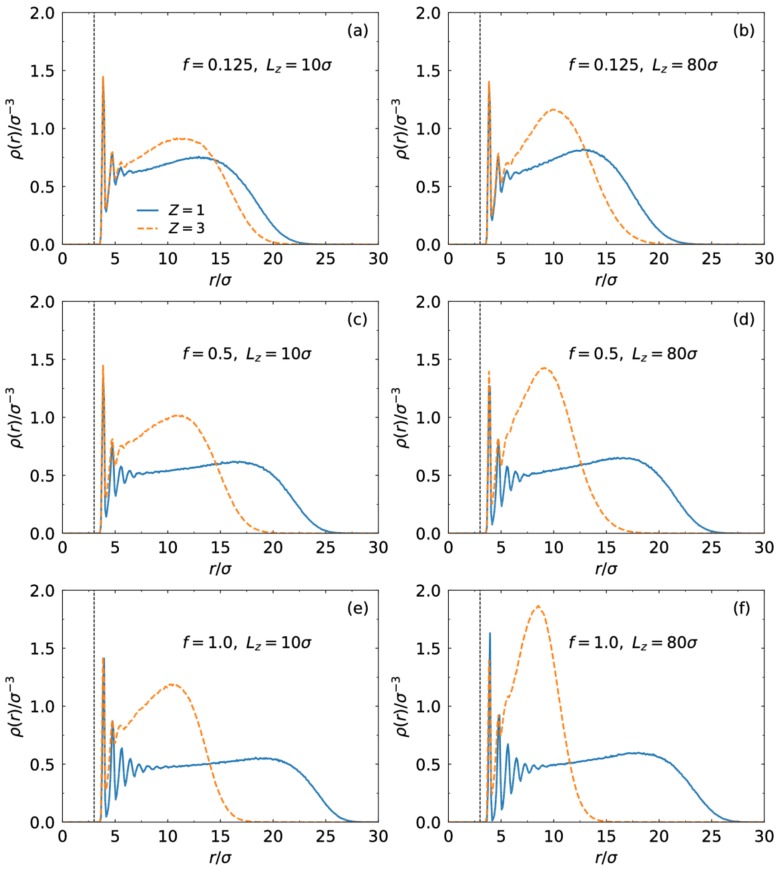
Monomer density of the SPB in the presence of monovalent (solid lines) or trivalent counterions (dashed lines) for different charge fraction and slit width.

In Figure 1, we present monomer density profiles as a function of distance from the center of the core for different charge fraction at $L_{z}=10\sigma$ and 80σ. At $L_{z}=10\sigma$, the SPB is confined strongly between two walls along the z direction. In the presence of monovalent counterions, it was found that as the charge fraction f increases, the chains extend far away from the center of the core. For the large slit width $L_{z}=80\sigma$, the interactions between the SPB and walls disappear. The increase of f also results in stretched chain conformations. For the monovalent cases, the electrostatic repulsion between charged monomers is enhanced with the increase of the charge fraction, which forces the chains to extend. However, for the trivalent cases the larger charge fraction corresponds to stronger counterion binding to charged monomers. The enhancement of trivalent counterion binding leads to collapsed brush conformation [20,37]. Our study indicates that the counterion binding effect overwhelms the wall compression in the present parameter setting. By comparison, for the trivalent cases the strong confinement at $L_{z}=10\sigma$ still leads to a relatively stretched brush conformation.

**Figure 2:**
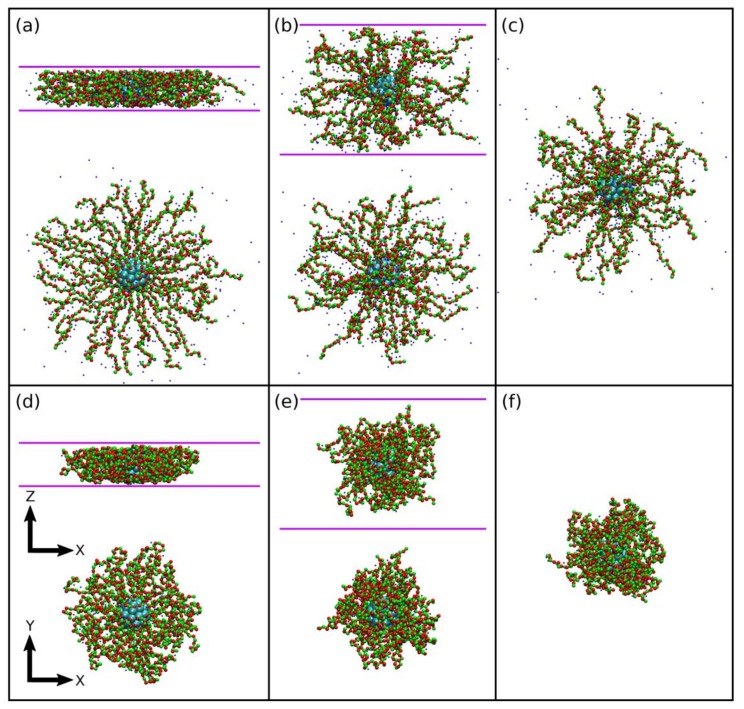
Typical simulation snapshots of SPBs with f=0.5 in the presence of (**a**–**c**) monovalent and (**d**–**f**) trivalent counterions at (**a**,**d**) $L_{z}=10\sigma$, (**b**,**e**) 30σ and (**c**,**f**) 80σ. The green and red beads represent charged and neutral monomers, respectively. The small blue beads are counterions. The cyan beads represent wall particles on the core. For the cases of (**a**,**d**) $L_{z}=10\sigma$ and (**b**,**e**) 30σ, side (xz plane) and top views (xy plane) are shown.

To obtain intuitive view of the brush system under confinement, we present typical snapshots of SPBs for different slit width and counterion valence in Figure 2. Almost all trivalent counterions owing to their strong binding to the charged chains are trapped into the brush (Figure 2d–f), but some monovalent counterions diffuse freely outside the brush (Figure 2a–c). The brush in the presence of trivalent counterions is in a collapsed state regardless of strong or weak confinement. Under the strong confinement along the z direction, the chains are squeezed to extend in the xy plane.

**Figure 3:**
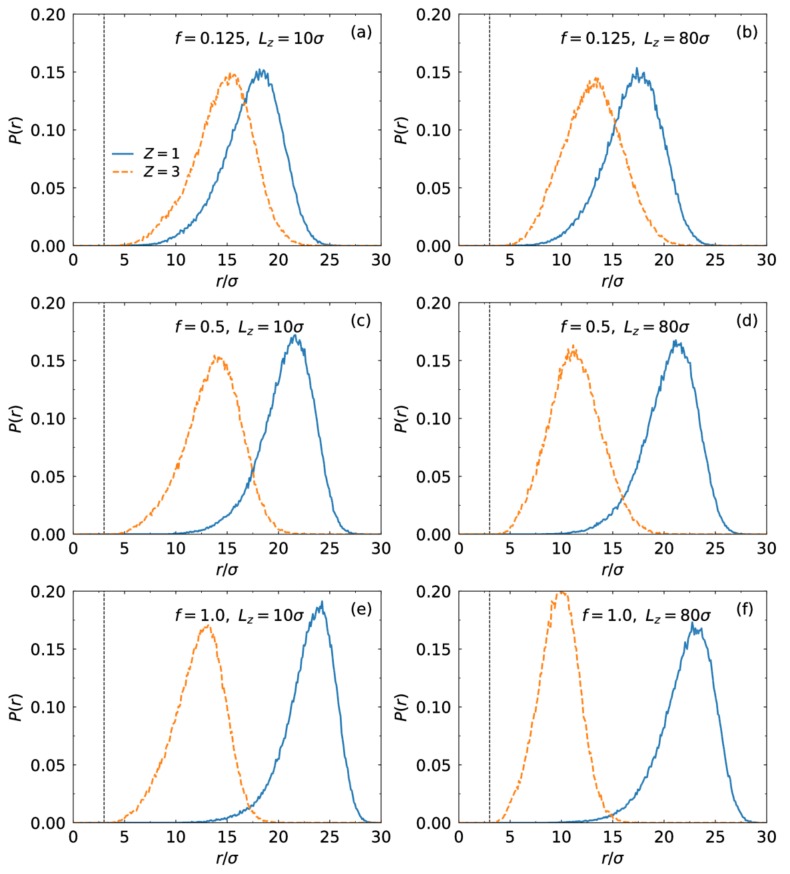
Distribution of end monomers as a function of distance from the center of the core in the presence of monovalent (solid lines) or trivalent counterions (dashed lines) for different charge fraction and slit width.

We present the distribution of end monomers for different charge fraction and slit width in Figure 3. Similar to the monomer density in Figure 1, the peak of the distribution of end monomers moves away from the core with the increase of the charge fraction for the monovalent case but is close to for the trivalent case. At f=1.0, most end monomers for the monovalent case distribute in the region away from the core. For the trivalent case, the chains fold back to the core strongly at $L_{z}=80\sigma$.

**Figure 4:**
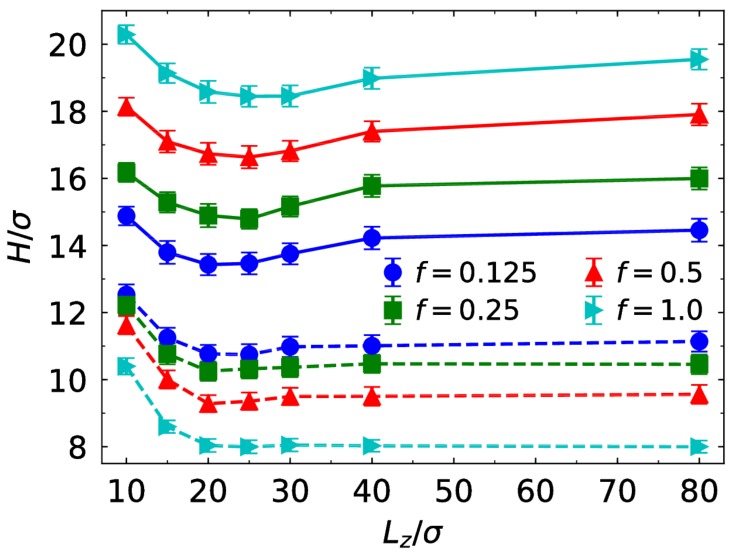
Brush thickness as a function of $L_{z}$ for different charge fraction f in the presence of monovalent (solid lines) and trivalent counterions (dashed lines).

We plot the brush thickness for different charge fractions as a function of $L_{z}$ in Figure 4. Here, we define the brush thickness as $H={\left[\langle {\left(R_{m}-R\right)}^{2}\rangle \right]}^{1/2}$ where $R_{m}$ is the distance of terminal monomers from the center of the core, and R is the radius of the core. At a fixed value of the slit width, increasing the charge fraction induces increased brush thickness for the monovalent case, but reduces the brush thickness for the trivalent case. As discussed above, the electrostatic binding effect becomes significantly strong for the trivalent case as the charge fraction increases. In addition, we also can find that the effect of the wall on the brush conformation is negligible at small $L_{z}$ for the trivalent case, especially at f=1.0 (the brush thickness keeps constant when $L_{z}$ extends 20σ). However, for the monovalent case the wall still influences the brush conformation at $L_{z}=40\sigma$ to some extent. We also observe that the brush thickness depends on the slit width nonlinearly. In particular, for the monovalent case, the brush thickness decreases with the increase of the slit width, until reaches a minimum, then increases. At small $L_{z}$, the steric repulsion in the brush is reduced as the slit width increases, leading to decreased brush thickness. At large slit width, the steric repulsion becomes very weak. The increase of the slit width does not contribute to steric repulsion, but some chains squeezed by the wall are extended. The minimum brush thickness appears with the decrease of the slit width when the compressed chains which contact with the walls begin to extend owing to enhanced steric repulsion. In the trivalent case, the brush is in a compact state especially for large charge fraction. As a result, the chains do not extend more largely when the slit width exceeds $L_{z}=20\sigma$.

**Figure 5:**
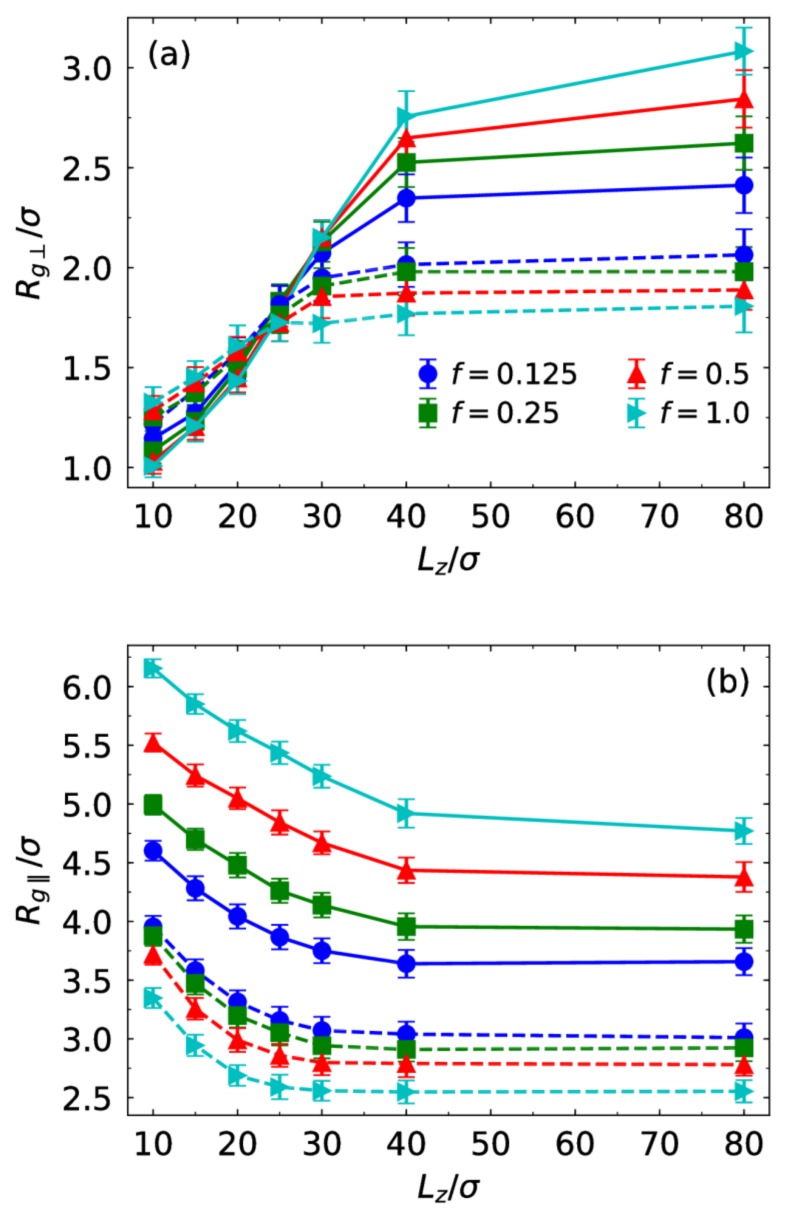
(**a**) Perpendicular component $R_{g⊥}$ and (**b**) parallel component $R_{g||}$ of the radius of gyration of the chains as a function of $L_{z}$ for different charge fraction f in the presence of monovalent (solid lines) and trivalent counterions (dashed lines).

We characterize the structural anisotropy of the brush due to the confinement in the z direction through calculating the perpendicular component $R_{g⊥}$ and parallel component $R_{g||}$ of the radius of gyration of the chains. $R_{g⊥}$ increases as the slit width increases (Figure 5a). At large slit width where the chains do not contact with the walls, the charge fraction plays a more important role in determining $R_{g⊥}$. For the monovalent case, $R_{g⊥}$ increases with the charge fraction but decreases for the trivalent case. As the confinement is enhanced, the effect of the charge fraction is weakened. The difference of $R_{g⊥}$ for different charge fractions becomes much smaller. We note that at $L_{z}<25\sigma$ the perpendicular component for the trivalent case is larger compared to the monovalent case. This is caused by strong extension of the chains in the xy plane in the presence of monovalent counterions at small slit width. The strong electrostatic binding of trivalent counterions to the chains results in smaller chain extension. The parallel component $R_{g||}$ decreases as the slit width increases (Figure 5b). The effect essentially disappears until there is no interaction between the chains and walls. At a fixed charge fraction, an obvious increase of the parallel component for the monovalent case occurs at larger slit width. For example, at f=1.0 $R_{g||}$ for the monovalent case increases evidently when $L_{z}<40\sigma$ but for the trivalent case a significant increase is observed until $L_{z}<25\sigma$.

### 3.2. Electrostatic Correlation and Counterion Diffusion

**Figure 6:**
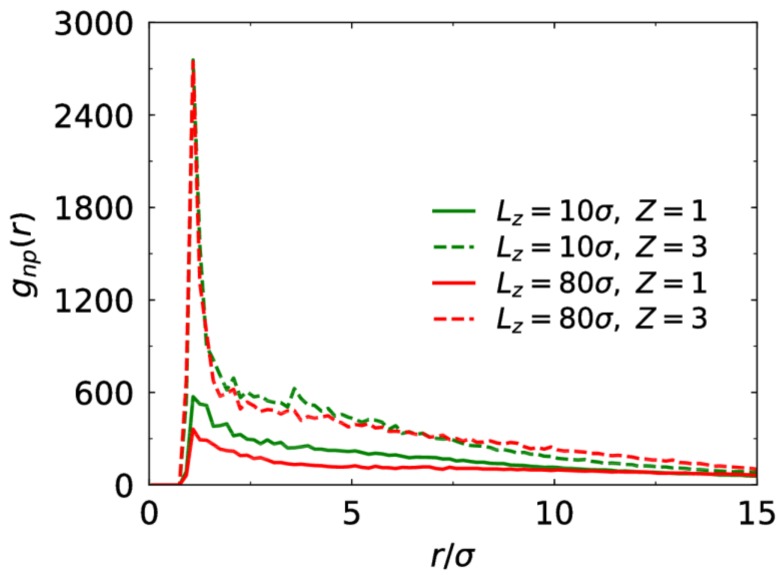
Radial distribution function between charged monomers and their counterions for different counterion valence and slit width at f=0.5.

Electrostatic correlation between charged monomers and counterions can be characterized by their radial distribution functions (RDFs). As seen in Figure 6, trivalent counterions have much stronger correlation with the chains compared to the monovalent counterions. This is demonstrated in many polyelectrolyte systems [20,38,39]. Here, we further study the effect of the confinement on the electrostatic correlation for the monovalent and trivalent cases. When the counterions are trivalent, the first peak of the RDF profiles is almost equal for $L_{z}=10\sigma$ and 80σ. It indicates that the confinement does not influence trivalent counterion-monomer correlation. However, we find that the correlation between monovalent counterions and charged monomers at strong confinement is stronger than at weak confinement. For the trivalent case, almost all counterions are bound to the brush, and thus the confinement does not influence the amount of counterions trapped in the brush. However, for the monovalent case, some counterions diffuse outside the brush. When the slit width is reduced, some monovalent counterions that diffuse freely enter the brush, leading to enhanced counterion-monomer correlation.

**Figure 7:**
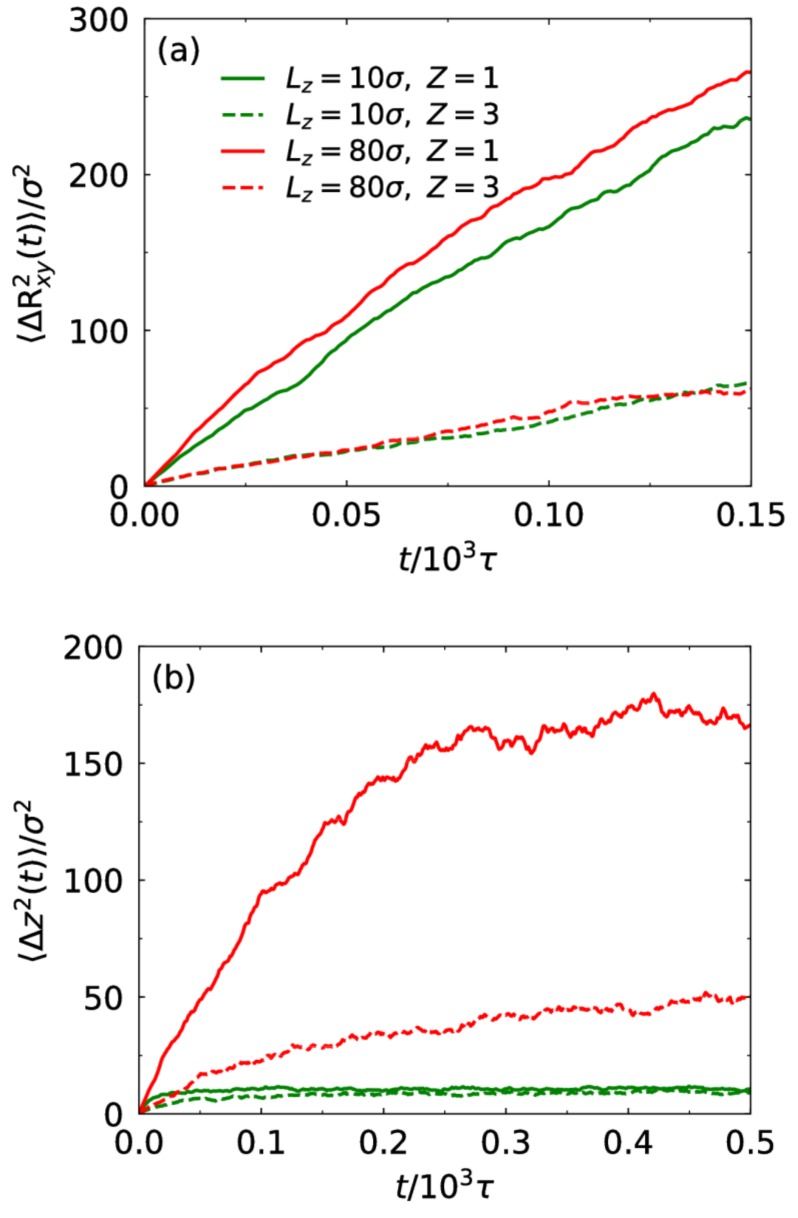
(**a**) The MSD $\Delta R_{xy}^{2}$ in the xy plane and (**b**) the z-directional MSD $\Delta z^{2}$ of counterions for different counterion valence and slit width at f=0.5.

Finally, we examine the mobility of counterions through their mean square displacement (MSD). The confinement effect leads to anisotropic diffusion of counterions. Here, we divide the diffusion into two components: lateral diffusion $\Delta R_{xy}^{2}$ in the xy plane and perpendicular diffusion $\Delta z^{2}$ along the z direction. Figure 7 presents these two components of monovalent and trivalent counterions at $L_{z}=10\sigma$ and 80σ. The mobility of counterions is strongly related to the correlation between them and the chains. For the lateral component, the mobility of the trivalent counterions is not influenced when the slit width is changed (Figure 7a). At $L_{z}=10\sigma$, the brush is largely compressed. Compared to the case of $L_{z}=10\sigma$, though the trivalent counterions is still confined in the brush at $L_{z}=80\sigma$, their range of motion along the direction normal to the wall is extended (Figure 7b). At $L_{z}=10\sigma$, more monovalent counterions are bound to the chains. This results in weaker mobility regardless of lateral or perpendicular components.

## 4. Conclusions

In this work, based on molecular dynamics simulations we investigated conformational behavior of the SPB in the presence of monovalent and trivalent counterions in a confined environment. The brush exhibits extended conformation under the confinement due to excluded-volume interactions. The brush thickness depends on the slit width nonlinearly and reaches minimum at intermediate slit width. It was found that the effect of the charge fraction on the brush conformation is opposite for the monovalent and trivalent counterion cases. In the presence of monovalent counterions, the brush thickness increases with the charge fraction. However, the grafted chains contract towards the core for the trivalent counterion case. For the trivalent case, the confinement does not influence the amount of counterions trapped in the brush because almost all counterions are bound to the brush. For the monovalent case, as the slit width decreases, some monovalent counterions that diffuse freely enter the brush, leading to enhanced counterion-monomer correlation. Additionally, the electrostatic correlation also affects the diffusion of counterions under the confinement.

## References

[1] Rühe J., Ballauff M., Biesalski M., Dziezok P., Grohn F., Johannsmann D., Houbenov N., Hugenberg N., Konradi R., Minko S. (2004). Polyelectrolyte brushes. Adv. Polym. Sci..

[2] Ballauff M., Borisov O. (2006). Polyelectrolyte brushes. Curr. Opin. Colloid Interface Sci..

[3] Ballauff M. (2007). Spherical polyelectrolyte brushes. Prog. Polym. Sci..

[4] Toomey R., Tirrell M. (2008). Functional polymer brushes in aqueous media from self-assembled and surface-initiated polymers. Annu. Rev. Phys. Chem..

[5] Mercurieva A.A., Birshtein T.M., Zhulina E.B., Iakovlev P., van Male J., Leermakers F.A.M. (2002). An annealed polyelectrolyte brush in a polar-nonpolar binary solvent: Effect of pH and ionic strength. Macromolecules.

[6] Israels R., Leermakers F.A.M., Fleer G.J., Zhulina E.B. (1994). Charged Polymeric Brushes: Structure and Scaling Relations. Macromolecules.

[7] Zhulina E.B., Borisov O.V., van Male J., Leermakers F.A.M. (2001). Adsorption of tethered polyelectrolytes onto oppositely charged solid-liquid interfaces. Langmuir.

[8] Pincus P. (1991). Colloid stabilization with grafted polyelectrolytes. Macromolecules.

[9] Csajka F.S., Netz R.R., Seidel C., Joanny J.F. (2001). Collapse of polyelectrolyte brushes: scaling theory and simulations. Eur. Phys. J. E.

[10] Borisov O.V., Birshtein T.M., Zhulina E.B. (1991). Collapse of grafted polyelectrolyte layer. J. Phys. II.

[11] Nap R., Gong P., Szleifer I. (2006). Weak polyelectrolytes tethered to surfaces: effect of geometry, acid-base equilibrium and electrical permittivity. J. Polym. Sci., Part B: Polym. Phys..

[12] Gong P., Genzer J., Szleifer I. (2007). Phase behavior and charge regulation of weak polyelectrolyte grafted layers. Phys. Rev. Lett..

[13] Gong P., Wu T., Genzer J., Szleifer I. (2007). Behavior of surface-anchored poly(acrylic acid) brushes with grafting density gradients on solid substrates: 2. Theory. Macromolecules.

[14] Csajka F.S., Seidel C. (2000). Strongly charged polyelectrolyte brushes: a molecular dynamics study. Macromolecules.

[15] Seidel C. (2003). Strongly stretched polyelectrolyte brushes. Macromolecules.

[16] Naji A., Netz R.R., Seidel C. (2003). Non-linear osmotic brush regime: Simulations and mean-field theory. Eur. Phys. J. E.

[17] Hehmeyer O.J., Arya G., Panagiotopoulos A.Z., Szleifer I. (2007). Monte Carlo simulation and molecular theory of tethered polyelectrolytes. J. Chem. Phys..

[18] Guptha V.S., Hsiao P.Y. (2014). Polyelectrolyte brushes in monovalent and multivalent salt solutions. Polymer.

[19] Yan L.T., Xu Y.Y., Ballauff M., Muller A.H.E., Boker A. (2009). Influence of Counterion Valency on the Conformational Behavior of Cylindrical Polyelectrolyte Brushes. J. Phys. Chem. B.

[20] Mei Y., Hoffmann M., Ballauff M., Jusufi A. (2008). Spherical polyelectrolyte brushes in the presence of multivalent counterions: The effect of fluctuations and correlations as determined by molecular dynamics simulations. Phys. Rev. E.

[21] Ni R., Cao D., Wang W., Jusufi A. (2008). Conformation of a Spherical Polyelectrolyte Brush in the Presence of Oppositely Charged Linear Polyelectrolytes. Macromolecules.

[22] Cao Q., Zuo C., Li L. (2011). Electrostatic binding of oppositely charged surfactants to spherical polyelectrolyte brushes. Phys. Chem. Chem. Phys..

[23] Cao Q.Q., Zuo C.C., Li L.J., Zhang Y.H. (2012). Modulation of electroosmotic flow by electric field-responsive polyelectrolyte brushes: A molecular dynamics study. Microfluid. Nanofluid..

[24] Cao Q.Q., Zuo C.C., Li L.J., Yan G. (2011). Effects of chain stiffness and salt concentration on responses of polyelectrolyte brushes under external electric field. Biomicrofluidics.

[25] Ouyang H., Xia Z.H., Zhe J. (2010). Voltage-controlled flow regulating in nanofluidic channels with charged polymer brushes. Microfluid. Nanofluid..

[26] Binder K., Horbach J., Vink R., De Virgiliis A. (2008). Confinement effects on phase behavior of soft matter systems. Soft Matter.

[27] Fritsche M., Heermann D.W. (2011). Confinement driven spatial organization of semiflexible ring polymers: Implications for biopolymer packaging. Soft Matter.

[28] Reisner W., Pedersen J.N., Austin R.H. (2012). DNA confinement in nanochannels: physics and biological applications. Rep. Prog. Phys..

[29] Bakajin O.B., Duke T.A.J., Chou C.F., Chan S.S., Austin R.H., Cox E.C. (1998). Electrohydrodynamic Stretching of DNA in Confined Environments. Phys. Rev. Lett..

[30] Javidpour L., Sahimi M. (2011). Confinement in nanopores can destabilize α-helix folding proteins and stabilize the β structures. J. Chem. Phys..

[31] Paturej J., Milchev A., Egorov S.A., Binder K. (2013). Star polymers confined in a nanoslit: a simulation test of scaling and self-consistent field theories. Soft Matter.

[32] Konieczny M., Likos C.N. (2006). Polyelectrolyte stars in planar confinement. J. Chem. Phys..

[33] Konieczny M., Likos C.N. (2007). From sea-urchins to starfishes: controlling the adsorption of star-branched polyelectrolytes on charged walls. Soft Matter.

[34] Cao Q., You H. (2017). Morphologies of spherical polyampholyte brushes: Effects of counterion valence and charged monomer sequence. Polymer.

[35] Kremer K., Grest G.S. (1990). Dynamics of entangled linear polymer melts: A molecular-dynamics simulation. J. Chem. Phys..

[36] Yeh I.-C., Berkowitz M.L. (1999). Ewald summation for systems with slab geometry. J. Chem. Phys..

[37] Mei Y., Lauterbach K., Hoffmann M., Borisov O.V., Ballauff M., Jusufi A. (2006). Collapse of Spherical Polyelectrolyte Brushes in the Presence of Multivalent Counterions. Phys. Rev. Lett..

[38] Solis F.J., Cruz M.O.d.l. (2000). Collapse of flexible polyelectrolytes in multivalent salt solutions. J. Chem. Phys..

[39] He S., Arscott P.G., Bloomfield V.A. (2000). Condensation of DNA by multivalent cations: Experimental studies of condensation kinetics. Biopolymers.

